# *catena*-Poly[[diaqua­nickel(II)]-μ-7-oxabicyclo­[2.2.1]heptane-2,3-di­carboxyl­ato]

**DOI:** 10.1107/S1600536809021771

**Published:** 2009-06-17

**Authors:** Yun-Yun Wang, Rui-Ding Hu, Wen-Zhong Zhu, Qiu-Yue Lin

**Affiliations:** a Zhejiang Key Laboratory for Reactive Chemistry on Solid Surfaces, Institute of Physical Chemistry, Zhejiang Normal University, Jinhua, Zhejiang 321004, People’s Republic of China, and, College of Chemistry and Life Science, Zhejiang Normal University, Jinhua 321004, Zhejiang, People’s Republic of China

## Abstract

In the crystal structure of the title compound, [Ni(C_8_H_8_O_5_)(H_2_O)_2_]_*n*_, the Ni^II^ cation is in a Jahn–Teller-distorted octahedral coordination environment binding to two O atoms from water molecules, the bridging O atom of the bicycloheptane unit, two carboxylate O atoms from different carboxylate groups and one carboxylate O atom from a symmetry-related bridging ligand. The crystal structure is made up from layers propagating parallel to the bc plane.

## Related literature

For the structure of the Cu(II) analogue, see: Wang *et al.* (2009 ▶).
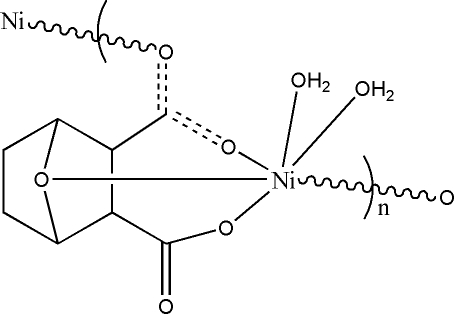


## Experimental

### 

#### Crystal data

[Ni(C_8_H_8_O_5_)(H_2_O)_2_]
*M* *_r_* = 278.89
Monoclinic, 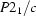

*a* = 10.9145 (2) Å
*b* = 8.6281 (2) Å
*c* = 10.8581 (2) Å
β = 107.351 (1)°
*V* = 975.99 (3) Å^3^
*Z* = 4
Mo *K*α radiation
μ = 2.01 mm^−1^
*T* = 296 K
0.27 × 0.20 × 0.10 mm

#### Data collection

Bruker APEXII area-detector diffractometer
Absorption correction: multi-scan (*SADABS*; Sheldrick, 1996 ▶) *T*_min_ = 0.618, *T*_max_ = 0.817
7972 measured reflections
2213 independent reflections
1961 reflections with *I* > 2σ(*I*)
*R*_int_ = 0.019

#### Refinement

*R*[*F* ^2^ > 2σ(*F* ^2^)] = 0.022
*wR*(*F* ^2^) = 0.059
*S* = 1.02
2213 reflections
157 parameters
6 restraints
H atoms treated by a mixture of independent and constrained refinement
Δρ_max_ = 0.30 e Å^−3^
Δρ_min_ = −0.30 e Å^−3^

### 

Data collection: *APEX2* (Bruker, 2006 ▶); cell refinement: *SAINT* (Bruker, 2006 ▶); data reduction: *SAINT*; program(s) used to solve structure: *SHELXS97* (Sheldrick, 2008 ▶); program(s) used to refine structure: *SHELXL97* (Sheldrick, 2008 ▶); molecular graphics: *SHELXTL* (Sheldrick, 2008 ▶); software used to prepare material for publication: *SHELXL97*.

## Supplementary Material

Crystal structure: contains datablocks I, global. DOI: 10.1107/S1600536809021771/at2793sup1.cif

Structure factors: contains datablocks I. DOI: 10.1107/S1600536809021771/at2793Isup2.hkl

Additional supplementary materials:  crystallographic information; 3D view; checkCIF report

## Figures and Tables

**Table 1:** Hydrogen-bond geometry (Å, °)

*D*—H⋯*A*	*D*—H	H⋯*A*	*D*⋯*A*	*D*—H⋯*A*
O1*W*—H1*WA*⋯O2^i^	0.841 (15)	2.062 (15)	2.9027 (19)	180 (3)
O1*W*—H1*WB*⋯O3^ii^	0.822 (15)	2.135 (18)	2.7953 (17)	137 (2)
O1*W*—H1*WB*⋯O1^iii^	0.822 (15)	2.366 (19)	3.1013 (17)	149 (2)
O2*W*—H2*WA*⋯O4^i^	0.824 (16)	1.888 (16)	2.6967 (19)	167 (3)
O2*W*—H2*WB*⋯O2^ii^	0.802 (16)	2.341 (16)	3.135 (2)	170 (2)

Symmetry codes: (i) 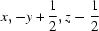; (ii) 
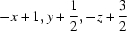
; (iii) 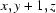.

**Fig. 1.**
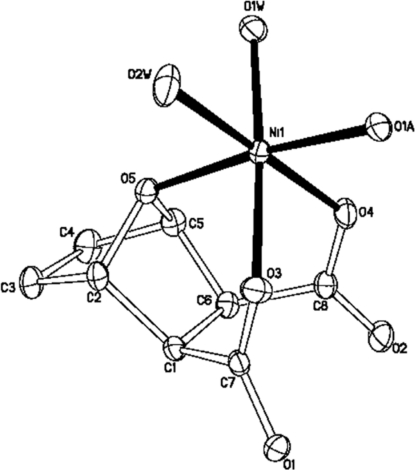
A view of the molecule of (I) showing the atom-labelling scheme with displacement ellipsoids drawn at the 30% probability.

## supplementary crystallographic information

### Comment

The title compound, (I), is isostructural with the Cu(II) analogue (Wang *et al.*, 2009). In the title compound, each Ni^II^ ion is six-coordinated by two oxygen atoms from water, one bridge oxygen, two carboxylate oxygen atoms in two different carboxylate groups and one carboxylate oxygen atom in another asymmetric unit. O1, O1W, O5 and O3 lie in the equatorial plane with the torsion angle -1.121 (47)°. O2W and carboxylate oxygen atom O4 are in the axial positions. The bond angle of O2W—Ni1—O4 is 177.144 (52)°, so it forms a distorted octahedral. Owing to the binding of the bridge oxygen atom with Ni, two six-membered rings(Ni1/O4/C8/C6/C5/O5 and Ni1/O3/C7/C1/C2/O5) are created. In addition, a seven-membered ring (Ni1/O3/C7/C1/C6/C8/O4) is formed because of the coordination of carboxylate oxygen atoms O3 and O4. What's more, intermolecular O—H···O hydrogen bonds of the complex make the crystal structure more stable (Table 1).

### Experimental

A mixture of 1 mmol norcantharidin, 1 mmol NiCl_2_.6H_2_O and 15 mL distilled water was sealed in a 25 mL Teflon-lined stainless vessel and heated at 443 K for 3 d, then cooled slowly to room temperature. The solution was filtered and block green crystals were obtained.

### Refinement

The H atoms bonded to C atoms were positioned geometrically and refined using a riding model [C—H = 0.97-0.98 Å, *U*_iso_(H) = 1.2*U*_eq_(C)]. The H atoms bonded to O atoms were located in a difference Fourier maps and refined with O—H distance restraints of 0.85 (2) and *U*_iso_(H) = 1.5*U*_eq_(O).

### Crystal data

**Table:**

[Ni(C_8_H_8_O_5_)(H_2_O)_2_]	*F*(000) = 576
*M**_r_* = 278.89	*D*_x_ = 1.898 Mg m^−^^3^
Monoclinic, *P*2_1_/*c*	Mo *K*α radiation, λ = 0.71073 Å
Hall symbol: -P 2ybc	Cell parameters from 4375 reflections
*a* = 10.9145 (2) Å	θ = 2.0–27.5°
*b* = 8.6281 (2) Å	µ = 2.01 mm^−^^1^
*c* = 10.8581 (2) Å	*T* = 296 K
β = 107.351 (1)°	Block, green
*V* = 975.99 (3) Å^3^	0.27 × 0.20 × 0.10 mm
*Z* = 4	

### Data collection

**Table:**

Bruker APEXII area-detector diffractometer	2213 independent reflections
Radiation source: fine-focus sealed tube	1961 reflections with *I* > 2σ(*I*)
graphite	*R*_int_ = 0.019
ω scans	θ_max_ = 27.5°, θ_min_ = 2.0°
Absorption correction: multi-scan (*SADABS*; Sheldrick, 1996)	*h* = −13→14
*T*_min_ = 0.618, *T*_max_ = 0.817	*k* = −11→6
7972 measured reflections	*l* = −12→14

### Refinement

**Table:**

Refinement on *F*^2^	Primary atom site location: structure-invariant direct methods
Least-squares matrix: full	Secondary atom site location: difference Fourier map
*R*[*F*^2^ > 2σ(*F*^2^)] = 0.022	Hydrogen site location: inferred from neighbouring sites
*wR*(*F*^2^) = 0.059	H atoms treated by a mixture of independent and constrained refinement
*S* = 1.02	*w* = 1/[σ^2^(*F*_o_^2^) + (0.032*P*)^2^ + 0.4246*P*] where *P* = (*F*_o_^2^ + 2*F*_c_^2^)/3
2213 reflections	(Δ/σ)_max_ = 0.001
157 parameters	Δρ_max_ = 0.30 e Å^−^^3^
6 restraints	Δρ_min_ = −0.30 e Å^−^^3^

### Special details

**Table:**

Geometry. All e.s.d.'s (except the e.s.d. in the dihedral angle between two l.s. planes) are estimated using the full covariance matrix. The cell e.s.d.'s are taken into account individually in the estimation of e.s.d.'s in distances, angles and torsion angles; correlations between e.s.d.'s in cell parameters are only used when they are defined by crystal symmetry. An approximate (isotropic) treatment of cell e.s.d.'s is used for estimating e.s.d.'s involving l.s. planes.
Refinement. Refinement of *F*^2^ against ALL reflections. The weighted *R*-factor *wR* and goodness of fit *S* are based on *F*^2^, conventional *R*-factors *R* are based on *F*, with *F* set to zero for negative *F*^2^. The threshold expression of *F*^2^ > σ(*F*^2^) is used only for calculating *R*-factors(gt) *etc*. and is not relevant to the choice of reflections for refinement. *R*-factors based on *F*^2^ are statistically about twice as large as those based on *F*, and *R*- factors based on ALL data will be even larger.

### Fractional atomic coordinates and isotropic or equivalent isotropic displacement parameters (Å^2^)

**Table:**

	*x*	*y*	*z*	*U*_iso_*/*U*_eq_	
C1	0.73787 (15)	−0.1019 (2)	0.71021 (15)	0.0214 (3)	
H1A	0.7710	−0.2053	0.7005	0.026*	
C2	0.75289 (16)	0.0067 (2)	0.60404 (16)	0.0234 (3)	
H2A	0.6816	0.0008	0.5240	0.028*	
C3	0.88388 (17)	−0.0131 (2)	0.58426 (19)	0.0329 (4)	
H3A	0.8888	0.0402	0.5072	0.039*	
H3B	0.9048	−0.1216	0.5789	0.039*	
C4	0.97214 (18)	0.0632 (3)	0.7078 (2)	0.0375 (5)	
H4A	1.0324	−0.0110	0.7599	0.045*	
H4B	1.0192	0.1498	0.6872	0.045*	
C5	0.87589 (15)	0.1175 (2)	0.77610 (17)	0.0265 (4)	
H5A	0.9073	0.2034	0.8363	0.032*	
C6	0.82547 (16)	−0.0207 (2)	0.83620 (16)	0.0234 (3)	
H6A	0.8966	−0.0891	0.8805	0.028*	
C7	0.59962 (14)	−0.1119 (2)	0.71114 (15)	0.0202 (3)	
C8	0.75095 (16)	0.0292 (2)	0.92900 (15)	0.0242 (4)	
O1W	0.69190 (12)	0.44064 (15)	0.74994 (13)	0.0289 (3)	
H1WA	0.711 (2)	0.473 (3)	0.6846 (18)	0.043*	
H1WB	0.643 (2)	0.505 (2)	0.765 (2)	0.043*	
O1	0.56501 (12)	−0.23749 (14)	0.74852 (12)	0.0244 (3)	
O2W	0.51314 (14)	0.29376 (19)	0.53124 (13)	0.0383 (3)	
H2WA	0.557 (2)	0.322 (3)	0.486 (2)	0.057*	
H2WB	0.4472 (17)	0.342 (3)	0.512 (2)	0.057*	
O2	0.75780 (15)	−0.05131 (17)	1.02529 (12)	0.0389 (3)	
O3	0.52811 (11)	0.00446 (14)	0.67765 (13)	0.0272 (3)	
O4	0.68270 (12)	0.15134 (16)	0.89954 (11)	0.0302 (3)	
O5	0.76514 (11)	0.15762 (14)	0.66735 (11)	0.0236 (3)	
Ni1	0.601796 (19)	0.22532 (2)	0.714683 (19)	0.01937 (8)	

### Atomic displacement parameters (Å^2^)

**Table:**

	*U*^11^	*U*^22^	*U*^33^	*U*^12^	*U*^13^	*U*^23^
C1	0.0213 (8)	0.0212 (8)	0.0245 (8)	0.0010 (6)	0.0109 (6)	−0.0017 (6)
C2	0.0216 (8)	0.0296 (9)	0.0221 (8)	−0.0007 (7)	0.0111 (6)	−0.0032 (7)
C3	0.0287 (9)	0.0413 (11)	0.0364 (10)	0.0019 (8)	0.0217 (8)	−0.0008 (8)
C4	0.0218 (9)	0.0503 (13)	0.0452 (11)	−0.0038 (8)	0.0172 (8)	0.0003 (10)
C5	0.0196 (8)	0.0318 (10)	0.0285 (9)	−0.0051 (7)	0.0080 (7)	−0.0037 (7)
C6	0.0194 (7)	0.0285 (9)	0.0225 (8)	0.0031 (7)	0.0066 (6)	0.0014 (7)
C7	0.0218 (8)	0.0217 (8)	0.0194 (8)	−0.0035 (6)	0.0098 (6)	−0.0058 (6)
C8	0.0240 (8)	0.0299 (9)	0.0181 (8)	−0.0004 (7)	0.0053 (6)	−0.0013 (7)
O1W	0.0290 (7)	0.0249 (7)	0.0357 (7)	0.0008 (5)	0.0145 (6)	−0.0014 (5)
O1	0.0236 (6)	0.0228 (6)	0.0306 (6)	−0.0026 (5)	0.0138 (5)	0.0003 (5)
O2W	0.0377 (8)	0.0566 (10)	0.0238 (7)	0.0112 (7)	0.0144 (6)	0.0103 (6)
O2	0.0555 (9)	0.0399 (8)	0.0258 (7)	0.0123 (7)	0.0187 (6)	0.0097 (6)
O3	0.0220 (6)	0.0209 (6)	0.0421 (7)	−0.0007 (5)	0.0145 (5)	−0.0011 (5)
O4	0.0364 (7)	0.0357 (8)	0.0218 (6)	0.0119 (6)	0.0137 (5)	0.0046 (5)
O5	0.0234 (6)	0.0249 (6)	0.0254 (6)	0.0001 (5)	0.0118 (5)	0.0019 (5)
Ni1	0.02056 (12)	0.02009 (13)	0.01992 (12)	0.00083 (8)	0.00979 (9)	0.00170 (8)

### Geometric parameters (Å, °)

**Table:**

C1—C7	1.514 (2)	C6—H6A	0.9800
C1—C2	1.532 (2)	C7—O1	1.254 (2)
C1—C6	1.580 (2)	C7—O3	1.257 (2)
C1—H1A	0.9800	C8—O2	1.239 (2)
C2—O5	1.460 (2)	C8—O4	1.275 (2)
C2—C3	1.517 (2)	O1W—Ni1	2.0834 (13)
C2—H2A	0.9800	O1W—H1WA	0.841 (15)
C3—C4	1.546 (3)	O1W—H1WB	0.822 (15)
C3—H3A	0.9700	O1—Ni1^i^	2.0027 (12)
C3—H3B	0.9700	O2W—Ni1	2.0255 (13)
C4—C5	1.529 (2)	O2W—H2WA	0.824 (16)
C4—H4A	0.9700	O2W—H2WB	0.802 (16)
C4—H4B	0.9700	O3—Ni1	2.0608 (12)
C5—O5	1.457 (2)	O4—Ni1	2.0393 (12)
C5—C6	1.538 (2)	O5—Ni1	2.0809 (11)
C5—H5A	0.9800	Ni1—O1^ii^	2.0027 (12)
C6—C8	1.533 (2)		
			
C7—C1—C2	111.72 (13)	C1—C6—H6A	110.1
C7—C1—C6	111.48 (13)	O1—C7—O3	124.24 (14)
C2—C1—C6	101.96 (13)	O1—C7—C1	116.61 (15)
C7—C1—H1A	110.5	O3—C7—C1	119.13 (15)
C2—C1—H1A	110.5	O2—C8—O4	123.98 (16)
C6—C1—H1A	110.5	O2—C8—C6	119.16 (16)
O5—C2—C3	102.07 (14)	O4—C8—C6	116.85 (14)
O5—C2—C1	101.93 (12)	Ni1—O1W—H1WA	110.9 (17)
C3—C2—C1	110.77 (14)	Ni1—O1W—H1WB	109.8 (17)
O5—C2—H2A	113.6	H1WA—O1W—H1WB	106.5 (19)
C3—C2—H2A	113.6	C7—O1—Ni1^i^	125.62 (11)
C1—C2—H2A	113.6	Ni1—O2W—H2WA	119.0 (17)
C2—C3—C4	101.47 (14)	Ni1—O2W—H2WB	122.3 (17)
C2—C3—H3A	111.5	H2WA—O2W—H2WB	109 (2)
C4—C3—H3A	111.5	C7—O3—Ni1	120.63 (11)
C2—C3—H3B	111.5	C8—O4—Ni1	123.69 (11)
C4—C3—H3B	111.5	C5—O5—C2	96.15 (13)
H3A—C3—H3B	109.3	C5—O5—Ni1	115.54 (9)
C5—C4—C3	102.17 (14)	C2—O5—Ni1	113.69 (9)
C5—C4—H4A	111.3	O1^ii^—Ni1—O2W	87.33 (6)
C3—C4—H4A	111.3	O1^ii^—Ni1—O4	90.36 (5)
C5—C4—H4B	111.3	O2W—Ni1—O4	177.14 (6)
C3—C4—H4B	111.3	O1^ii^—Ni1—O3	82.07 (5)
H4A—C4—H4B	109.2	O2W—Ni1—O3	91.92 (6)
O5—C5—C4	101.75 (14)	O4—Ni1—O3	86.09 (5)
O5—C5—C6	102.27 (13)	O1^ii^—Ni1—O5	172.30 (5)
C4—C5—C6	110.67 (16)	O2W—Ni1—O5	91.84 (5)
O5—C5—H5A	113.7	O4—Ni1—O5	90.22 (5)
C4—C5—H5A	113.7	O3—Ni1—O5	90.31 (5)
C6—C5—H5A	113.7	O1^ii^—Ni1—O1W	103.17 (5)
C8—C6—C5	112.87 (15)	O2W—Ni1—O1W	88.95 (6)
C8—C6—C1	113.00 (13)	O4—Ni1—O1W	93.22 (5)
C5—C6—C1	100.21 (13)	O3—Ni1—O1W	174.73 (5)
C8—C6—H6A	110.1	O5—Ni1—O1W	84.46 (5)
C5—C6—H6A	110.1		
			
C7—C1—C2—O5	85.11 (15)	C6—C8—O4—Ni1	28.8 (2)
C6—C1—C2—O5	−34.06 (14)	C4—C5—O5—C2	56.04 (15)
C7—C1—C2—C3	−166.92 (14)	C6—C5—O5—C2	−58.43 (14)
C6—C1—C2—C3	73.92 (16)	C4—C5—O5—Ni1	176.00 (11)
O5—C2—C3—C4	35.96 (17)	C6—C5—O5—Ni1	61.53 (14)
C1—C2—C3—C4	−71.93 (18)	C3—C2—O5—C5	−57.44 (14)
C2—C3—C4—C5	−1.3 (2)	C1—C2—O5—C5	57.12 (13)
C3—C4—C5—O5	−33.65 (19)	C3—C2—O5—Ni1	−178.84 (10)
C3—C4—C5—C6	74.44 (18)	C1—C2—O5—Ni1	−64.27 (13)
O5—C5—C6—C8	−84.19 (16)	C8—O4—Ni1—O1^ii^	133.87 (14)
C4—C5—C6—C8	168.06 (15)	C8—O4—Ni1—O2W	97.8 (11)
O5—C5—C6—C1	36.27 (15)	C8—O4—Ni1—O3	51.85 (14)
C4—C5—C6—C1	−71.48 (16)	C8—O4—Ni1—O5	−38.45 (14)
C7—C1—C6—C8	−0.2 (2)	C8—O4—Ni1—O1W	−122.91 (14)
C2—C1—C6—C8	119.17 (15)	C7—O3—Ni1—O1^ii^	−141.13 (13)
C7—C1—C6—C5	−120.54 (14)	C7—O3—Ni1—O2W	131.83 (12)
C2—C1—C6—C5	−1.20 (15)	C7—O3—Ni1—O4	−50.22 (12)
C2—C1—C7—O1	149.60 (15)	C7—O3—Ni1—O5	39.98 (12)
C6—C1—C7—O1	−97.04 (17)	C7—O3—Ni1—O1W	32.4 (6)
C2—C1—C7—O3	−31.8 (2)	C5—O5—Ni1—O1^ii^	−105.7 (4)
C6—C1—C7—O3	81.59 (18)	C2—O5—Ni1—O1^ii^	4.1 (4)
C5—C6—C8—O2	−145.66 (17)	C5—O5—Ni1—O2W	170.59 (12)
C1—C6—C8—O2	101.50 (19)	C2—O5—Ni1—O2W	−79.56 (11)
C5—C6—C8—O4	35.8 (2)	C5—O5—Ni1—O4	−11.38 (12)
C1—C6—C8—O4	−77.0 (2)	C2—O5—Ni1—O4	98.46 (11)
O3—C7—O1—Ni1^i^	−8.3 (2)	C5—O5—Ni1—O3	−97.47 (11)
C1—C7—O1—Ni1^i^	170.29 (10)	C2—O5—Ni1—O3	12.37 (10)
O1—C7—O3—Ni1	145.58 (13)	C5—O5—Ni1—O1W	81.83 (12)
C1—C7—O3—Ni1	−32.95 (18)	C2—O5—Ni1—O1W	−168.33 (11)
O2—C8—O4—Ni1	−149.69 (15)		

Symmetry codes: (i) −*x*+1, *y*−1/2, −*z*+3/2; (ii) −*x*+1, *y*+1/2, −*z*+3/2.

### Hydrogen-bond geometry (Å, °)

**Table:**

*D*—H···*A*	*D*—H	H···*A*	*D*···*A*	*D*—H···*A*
O1W—H1WA···O2^iii^	0.84 (2)	2.06 (2)	2.9027 (19)	180 (3)
O1W—H1WB···O3^ii^	0.82 (2)	2.13 (2)	2.7953 (17)	137 (2)
O1W—H1WB···O1^iv^	0.82 (2)	2.37 (2)	3.1013 (17)	149 (2)
O2W—H2WA···O4^iii^	0.82 (2)	1.89 (2)	2.6967 (19)	167 (3)
O2W—H2WB···O2^ii^	0.80 (2)	2.34 (2)	3.135 (2)	170 (2)

Symmetry codes: (iii) *x*, −*y*+1/2, *z*−1/2; (ii) −*x*+1, *y*+1/2, −*z*+3/2; (iv) *x*, *y*+1, *z*.
